# Continuity of Care Across Hospital-to-Community Transitions: A Narrative Review Integrating Concepts, Measurement, and Nursing-Relevant Approaches

**DOI:** 10.3390/healthcare14050656

**Published:** 2026-03-05

**Authors:** Liron Markovich, Yael Sela, Keren Grinberg

**Affiliations:** 1 Department of Health Systems Management, Ariel University, Ariel 4070000, Israel; lironmarkovitch@gmail.com; 2 Department of Nursing Sciences, Faculty of Social and Community Sciences, Ruppin Academic Center, Emek Hefer 4025000, Israel; kereng@ruppin.ac.il

**Keywords:** continuity of care, care transitions, transitional care, care coordination, discharge planning, patient experience, integrated care

## Abstract

**Highlights:**

**What are the main findings?**

- Continuity of care during hospital-to-community transitions is a multidimensional construct encompassing informational, management, and relational continuity.
- Persistent gaps in information transfer and unclear professional accountability remain key contributors to fragmented transitions and negative patient experiences, particularly among older adults and people with complex conditions.

**What are the implications of the main findings?**

- Strengthening continuity requires coordinated, multi-level strategies that combine structured transitional processes with sustained professional relationships, especially through nursing roles.
- Evaluating continuity should integrate patient-reported measures with administrative and clinical indicators to capture whether care is experienced as coherent, supportive, and person-centered.

**Abstract:**

**Background:** Continuity of care is a core component of high-quality, patient-centered health systems and a central domain of nursing practice, particularly for older adults and people living with chronic and complex conditions. Yet discontinuities remain common during transitions between hospital and community care, contributing to fragmented communication, delayed follow-up, negative patient experiences, and avoidable harm. **Methods:** Literature was identified through iterative searches in PubMed and CINAHL (2002–2024), complemented by citation tracking of seminal frameworks and reference-list screening. Sources were prioritized for conceptual frameworks and empirical studies/reviews addressing hospital-to-community transitions, patient experience, and nursing-relevant strategies to strengthen continuity. **Results:** Across the reviewed literature, continuity was most frequently conceptualized as informational, management, and relational continuity. Most empirical studies and reviews highlighted discharge information-transfer failures and unclear post-discharge responsibility as recurrent drivers of discontinuity, particularly among older adults and people with complex needs. Evidence also suggests that interventions combining structured discharge processes with proactive post-discharge follow-up and a consistent point of contact (often nurse-led) are associated with improved patient experience and fewer early post-discharge complications in high-risk groups. Patient-reported instruments (e.g., PCCQ and CAHPS-derived domains) complement administrative indicators by capturing continuity as lived experience. Limitations: As a narrative review, findings reflect interpretative synthesis rather than systematic evidence aggregation. **Conclusions:** Continuity of care should be understood as both a structural and relational process; strengthening it likely requires multi-level strategies that address information transfer, accountability, and sustained therapeutic relationships across care transitions.

## 1. Introduction

Continuity of care has been described as a fundamental component of high-quality, patient-centered healthcare and a core attribute of strong primary and community-based health systems. In nursing practice across settings, continuity is not only a structural characteristic of care delivery but also a relational and professional commitment that supports safety, trust, and therapeutic effectiveness over time. This review also summarizes key evidence identified through iterative, non-systematic database and targeted citation searches to support health service and nursing practice implications.

As populations age and the prevalence of chronic and multimorbid conditions increases, more patients require care from multiple providers, disciplines, and organizations. This makes continuity both increasingly complex and increasingly important.

Transitions between hospital and community care represent a particularly vulnerable point in the patient journey. These transitions involve changes in care teams, treatment plans, and responsibility for follow-up, and they are often accompanied by gaps in communication, unclear accountability, and fragmented information. Research has linked such discontinuities to medication discrepancies, delayed follow-up, duplicated investigations, preventable complications, and negative patient experiences [1,2,3,4].

In routine nursing work, continuity is closely tied to core professional responsibilities in discharge planning, care coordination, patient education, and ongoing monitoring in the community. When continuity is weak, the burden of fragmentation is frequently shifted to patients and informal caregivers, who may need to navigate complex systems with limited guidance.

Continuity challenges are also shaped by health system context. In settings characterized by geographic, organizational, or workforce disparities (for example, rural or peripheral regions), access to services and timely follow-up may be limited, further complicating efforts to sustain coherent care over time [5]. In such contexts, strengthening continuity is both a quality and an equity priority.

Despite a substantial literature on discharge interventions and transitional care, gaps remain in conceptual syntheses that integrate (i) continuity of care as a multidimensional construct, (ii) patient experience and patient-reported measurement, and (iii) nursing-relevant perspectives across diverse health-system contexts. The aim of this narrative review is to clarify conceptualizations of continuity of care and synthesize evidence regarding its mechanisms, measurement approaches, and nursing-relevant strategies to strengthen continuity during hospital-to-community transitions. While previous reviews have addressed individual aspects of care transitions, this review uniquely integrates conceptual dimensions, measurement approaches, and intervention mechanisms, with a specific focus on the role of nursing. By synthesizing these elements together, the manuscript highlights both theoretical and practical implications that have not been systematically articulated in prior literature.

## 2. Materials and Methods

This narrative review draws on influential conceptual and empirical literature addressing continuity of care, care transitions, and related constructs in hospital and community settings. The purpose was to integrate and interpret central themes and perspectives to clarify concepts, highlight mechanisms, and discuss implications for nursing practice and patient experience across hospital-to-community transitions.

Literature identification and scope. Iterative, non-systematic searches were conducted in PubMed and CINAHL (2002–2024) using combinations of keywords related to continuity of care and transitions (e.g., continuity of care, care transitions, discharge planning, transitional care, care coordination, patient experience, patient-reported measures). Searches were complemented by reference-list screening and citation tracking to identify seminal and highly cited conceptual frameworks and additional relevant studies. Searches were scoped to reflect the publication years represented in the evidence base included in this review (2002–2024).

To improve transparency, sources were screened at the title/abstract and full-text level and prioritized for narrative synthesis when they provided widely used conceptual frameworks, empirical evidence on hospital-to-community transitions, or patient-reported measurement approaches.

Eligibility and prioritization. English-language conceptual papers, empirical studies (including trials and observational studies), and systematic/scoping reviews were eligible if they addressed continuity of care, care transitions, or related constructs in hospital-to-community contexts. Opinion pieces without substantive conceptual or empirical contribution were excluded. Priority was given to: (a) widely cited conceptual frameworks defining continuity and its dimensions; (b) empirical studies, trials, and reviews focusing on transitions of care- particularly among older adults and people living with chronic or complex conditions; and (c) articles addressing patient perspectives and measurement approaches, including patient-reported instruments relevant to the hospital-to-community transition. The initial searches identified approximately 34 studies. After applying eligibility criteria and prioritizing works for conceptual relevance, methodological rigor, and nursing-related insights, 20 studies were included in this narrative review, encompassing conceptual frameworks, empirical studies, and systematic/scoping reviews.

Data charting and synthesis approach. For included sources, we charted key characteristics (e.g., setting/population, focal construct or intervention, continuity dimension(s) addressed, and practice-relevant implications) and synthesized findings narratively. Because this is a narrative (rather than systematic) review, the synthesis does not aim to be exhaustive; instead, it emphasizes conceptual integration and practice-relevant interpretation. Where evidence was heterogeneous in terms of populations, settings, definitions, and outcomes, findings were presented as thematic patterns rather than pooled quantitative estimates.

Quality appraisal. A formal risk-of-bias appraisal was not undertaken because the review aim was conceptual and integrative and the included evidence was methodologically heterogeneous (spanning conceptual frameworks, trials, cross-sectional studies, and systematic/scoping reviews), which also precluded quantitative synthesis. To mitigate this limitation, we prioritized seminal and methodologically robust sources (e.g., highly cited frameworks and peer-reviewed empirical studies and reviews) and focused on consistency of themes across different study types.

Evidence strength considerations. Although a formal risk-of-bias tool was not applied, we considered the hierarchy and typical limitations of evidence when interpreting findings. Specifically, greater inferential weight was given to systematic/scoping reviews and controlled trials; moderate weight to well-described observational studies; and contextual weight to conceptual frameworks that clarify definitions and mechanisms. Throughout the results and discussion, we therefore use calibrated language (e.g., “several studies”, “most evidence suggests”, “evidence is mixed”) to reflect variation in study designs, outcomes, and contexts.

## 3. Results

### 3.1. Conceptualizing Continuity of Care

Continuity of care is a complex, multidimensional concept discussed across medicine, nursing, health services research, and policy. A highly influential multidisciplinary review by Haggerty and colleagues (2003) [6] defined continuity as the degree to which a series of discrete healthcare events is experienced by patients as coherent, connected, and consistent with their medical needs and personal context. This definition highlights continuity as both a system property and a patient experience constructed over time. More recent conceptual syntheses have further refined this framework, emphasizing the dynamic interaction between informational, management, and relational continuity across complex care transitions [7].

Haggerty et al. (2003) [6] proposed three interrelated dimensions of continuity that remain widely used in research and practice:

**Informational continuity**: The availability and use of relevant information on past events, personal circumstances, and patient preferences to make current care appropriate. In transitions, this includes complete and timely transfer of discharge summaries, medication lists, pending test results, and follow-up plans.

**Management continuity**: A consistent and coherent approach to managing a health condition that is responsive to a patient’s changing needs. This is particularly important for chronic and complex conditions requiring coordinated input from multiple professionals over time.

**Relational continuity**: An ongoing therapeutic relationship between a patient and one or more providers. In nursing and primary care, this is often expressed through trust, mutual knowledge, and a sense of responsibility and accountability that extends beyond single encounters.

These dimensions operate dynamically during transitions, where failures in one domain may disrupt the others. They underscore that continuity is not synonymous with any single intervention or organizational arrangement. Rather, it emerges from the interaction between information systems, organizational processes, professional roles, and interpersonal relationships. For nurses, continuity is enacted through formal mechanisms (such as care plans, discharge procedures, and follow-up protocols) and through sustained relationships with patients and families.

Continuity is related to, but distinct from, overlapping concepts such as discharge planning, care coordination, transitional care, and integrated care [7]. Table 1 summarizes these terms and highlights how each may contribute to continuity.

### 3.2. Why Continuity Matters: Mechanisms and Outcomes

Continuity of care influences patient outcomes and experiences through interrelated mechanisms that are particularly salient during transitions between hospital and community settings.

Communication and information transfer: Breakdowns in communication between hospital-based teams and community providers are among the most consistently documented problems in transitional care. Kripalani et al. (2007) [1] described substantial deficits in the timeliness, completeness, and accuracy of information transferred at discharge, including missing test results, unclear follow-up plans, and discrepancies in medication lists. Such failures undermine informational continuity and increase the risk of duplication, delays, and avoidable harm.

Patient safety and clinical outcomes: Continuity of care has been associated with improved outcomes in several settings; however, associations with endpoints such as mortality should be interpreted cautiously given confounding by baseline complexity, access to care, socioeconomic factors, and health system resources and because effects may differ by continuity dimension and study design [8]. Transitional care interventions designed to strengthen continuity have demonstrated improved outcomes for high-risk older adults, including reductions in rehospitalization and better post-discharge management [3,9].

Patient experience, trust, and therapeutic relationships: Continuity is a central determinant of patient experience. Patient experience reflects whether care is perceived as coordinated, respectful, understandable, and responsive to individual needs and has been associated with improved clinical safety and effectiveness outcomes [10,11]. Relational continuity supports trust and engagement in self-management and can reduce anxiety during vulnerable transition periods.

System-level implications: At the system level, poor continuity contributes to inefficiency, duplication of services, and potentially avoidable acute-care utilization. In contrast, systems that prioritize continuity and coordination, especially for people with complex needs, are better positioned to deliver effective and person-centered care [12].

### 3.3. Measuring Continuity and Patient Experience

Continuity can be measured from the perspectives of professionals, health systems, and patients. Administrative and provider-based indicators (for example, continuity indices derived from visit patterns) provide information about service utilization but capture only limited aspects of how continuity is experienced.

Because continuity is fundamentally longitudinal and relational, patient-reported measures are particularly important. Patient-reported instruments can assess whether care was perceived as coherent, coordinated, and supportive, complementing outcomes such as readmissions or emergency department use.

One instrument specifically developed to assess patients’ perceptions of continuity across the hospital-to-community transition is the Patient Continuity of Care Questionnaire (PCCQ). Hadjistavropoulos et al. (2008) [13] reported acceptable reliability and validity, capturing key elements of discharge and post-discharge processes including information transfer, coordination, and preparedness for self-management.

Broader patient experience surveys (for example, CAHPS-based tools) often include domains that overlap with continuity, such as care coordination and communication [10,14]. For quality improvement, combining patient-reported measures with clinical and administrative indicators provides a more complete understanding of whether care processes occurred and whether they were experienced as meaningful and supportive. Importantly, measurement approaches capture different continuity dimensions. Administrative indices (e.g., provider concentration or usual provider measures) may approximate longitudinal contact patterns and therefore relate most closely to relational continuity, but they do not directly assess informational quality or patients’ perceived coordination. Patient-reported measures can better capture whether transitions are experienced as coherent and responsive (e.g., clarity of discharge information, perceived coordination, and preparedness), yet they are sensitive to survey timing, recall and response bias, and potential ceiling effects. In hospital-to-community transitions, combining administrative indicators with patient-reported domains related to communication, coordination, and transition preparedness can provide a more balanced assessment, while acknowledging known measurement limitations and context-related differences (including equity-related differences in response patterns).

### 3.4. Vulnerable Populations and High-Risk Transitions

Continuity challenges are especially pronounced among older adults and individuals living with multiple chronic conditions. These patients often receive care from numerous professionals across settings over extended periods, increasing the likelihood of fragmented information, inconsistent management plans, and unclear responsibility for follow-up.

Older adults with complex needs may experience polypharmacy, functional limitations, cognitive impairment, and social vulnerability, all of which can complicate discharge planning and post-discharge management. System constraints can further exacerbate these risks. For example, delayed transfers of care have been associated with prolonged hospital stays and adverse outcomes among older patients [15]. Although much transitional-care evidence focuses on older adults and chronic conditions, discontinuities also affect other groups. Younger patients with complex needs (e.g., multi-morbidity, disability, or social vulnerability) may experience similar breakdowns in information transfer and accountability. Continuity challenges are also prominent in mental health transitions, where changes in medication, follow-up responsibility, and service boundaries (inpatient–community) can heighten relapse risk and undermine trust if relational continuity and timely follow-up are not maintained. In rural or peripheral settings, discontinuities may be amplified by limited service availability, longer travel distances, and longer wait times for follow-up, increasing the importance of clearly assigned accountability and proactive outreach after discharge. These context modifiers can affect the feasibility and “dose” of transitional care components and may shape which continuity dimension is most vulnerable (e.g., delayed follow-up affecting management continuity, limited access to a consistent contact affecting relational continuity).

From a nursing perspective, high-risk transitions often require intensive coordination, education, and monitoring in the community. When continuity is weak, patients and families may be left to navigate complex systems with insufficient guidance, increasing the likelihood of unmet needs and avoidable complications.

Health system characteristics, including resource constraints, workforce shortages, and geographic disparities, shape the risk of discontinuity. In rural or peripheral settings, limited access to specialized services and timely follow-up can intensify continuity challenges, making proactive coordination and strong community-based nursing services particularly important [5].

### 3.5. Intervention Categories and Evidence Patterns

Interventions described in the reviewed literature can be grouped into (i) discharge-focused strategies (e.g., standardized discharge summaries, medication reconciliation), (ii) post-discharge follow-up and outreach (often nurse-involved), (iii) navigation/case-management roles, (iv) transitional care programs combining multiple components, and (v) broader integrated-care or primary-care models that may enable continuity across settings. Across these categories, evidence is heterogeneous by population risk, intervention intensity (“dose”), implementation fidelity, and system context. Accordingly, we summarize recurrent patterns and plausible mechanisms while using calibrated language (e.g., “evidence suggests”, “findings are mixed”) to reflect variability in designs and outcomes.

### 3.6. Transitional Care Programs

Transitional care models are among the most extensively studied approaches to improving continuity across care settings. Programs commonly include structured discharge planning, patient and caregiver education, medication reconciliation, and proactive follow-up after discharge.

Nurse-led transitional care interventions for high-risk older adults have been associated with improved post-discharge outcomes and reduced rehospitalization [3]. Coleman’s Care Transitions Intervention emphasizes patient empowerment, improved self-management, and continuity of information across settings [2,9].

Across intervention studies and reviews, the strength and consistency of evidence varies by outcome, population, and implementation context. While several nurse-involved transitional care models report improvements in post-discharge preparedness and patient experience, effects on utilization outcomes (e.g., readmissions) are more heterogeneous across settings and risk strata. Differences in intervention “dose” (intensity and duration), role clarity, and integration with community services likely contribute to mixed findings. Accordingly, conclusions in this review emphasize patterns that recur across multiple study types while acknowledging outcome-specific variability.

### 3.7. Navigation and Care Coordination Roles

Navigation and care coordination can support patients, particularly older adults and those with chronic illness, in moving through complex systems. Manderson et al. (2012) [16] found that navigation roles may facilitate access to services, improve coordination between providers, and support patients during transitions.

These roles are often undertaken by nurses or other health professionals who act as consistent points of contact, strengthening management continuity and, when sustained over time, relational continuity.

### 3.8. Integrated Care Approaches

Integrated care frameworks aim to reduce fragmentation by improving coordination and collaboration across organizational and professional boundaries. They have been conceptualized as multilevel integration across clinical, professional, organizational, and system dimensions [17]. Gröne and Garcia-Barbero (2002) [12] described integrated care as a response to increasing system complexity and the needs of patients who require services from multiple providers over time. While integrated care is a broad policy and organizational concept, its objectives align closely with continuity: ensuring that care is experienced as coherent, connected, and person-centered.

### 3.9. Patient-Centered Models in Primary and Community Care

The Patient-Centered Medical Home (PCMH) is best understood here as an enabling primary-care context that may support post-discharge continuity (particularly management and relational continuity), rather than as a transition-specific intervention; direct evidence linking PCMH implementation to hospital-to-community transition outcomes may therefore vary across settings. Primary care-based models, including the PCMH, have been promoted as mechanisms to improve continuity, coordination, and quality of care. The PCMH emphasizes team-based care, proactive population management, enhanced access, and sustained relationships between patients and care teams [18].

For nurses working in primary and community settings, such models can provide structures that support ongoing follow-up, coordination with other services, and long-term therapeutic relationships.

**Figure 1:**
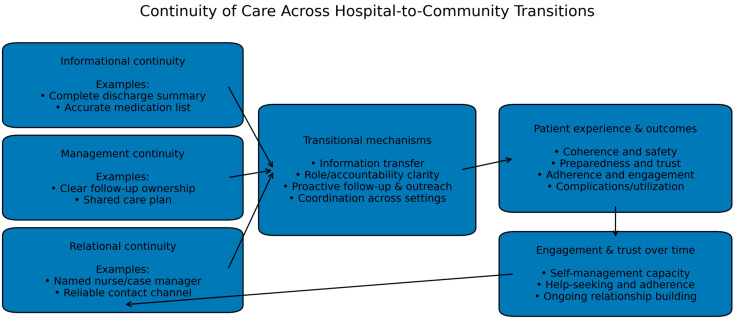
Continuity of Care Across Hospital-to-Community Transitions. The model illustrates how informational, management, and relational continuity influence transitional mechanisms (e.g., discharge information transfer, clear follow-up ownership, and a consistent point of contact) and shape patient experience and outcomes. Feedback loops indicate that patient experience and outcomes may influence subsequent engagement and trust, thereby affecting continuity over time.

Figure 1 presents a conceptual model summarizing how the three continuity dimensions influence mechanisms during care transitions and, in turn, patient and system outcomes.

## 4. Discussion

This review highlights continuity of care as a foundational component of high-quality, patient-centered healthcare, particularly during transitions between hospital and community settings. Continuity of care should be distinguished from related constructs such as care coordination; while coordination emphasizes the organization of tasks across providers, continuity focuses on sustained patient–provider relationships and consistent knowledge over time. The framework proposed by Haggerty et al. (2003) [6] remains useful for clinical and organizational improvement because informational, management, and relational continuity are interdependent; strengthening only one dimension in isolation is unlikely to substantially reduce fragmentation.

From a nursing perspective, a consistent pattern is the central role of nurses in operationalizing continuity through discharge planning, education, medication reconciliation, follow-up, and coordination across settings. Nurse-led and nurse-involved transitional care models illustrate that continuity is enacted not only through protocols but also through sustained professional relationships and clear clinical accountability [3,9,19]. Systematic reviews indicate that structured transitional care interventions may improve patient outcomes and reduce adverse events following discharge, particularly among older adults and patients with complex care needs [20].

A recurring issue across studies is vulnerability of information transfer at discharge. Despite longstanding awareness, deficits in completeness and timeliness of communication between hospital-based and community providers remain common [1]. Technical solutions such as electronic records and standardized forms are necessary but may be insufficient without explicit role clarity, ownership of follow-up, and shared expectations across settings.

The close relationship between continuity and patient experience also emerges clearly. Measures of patient experience capture coordination, respect, clarity, and responsiveness, which closely mirror elements of continuity [10,13]. Relational continuity can support trust and engagement in self-management and help identify emerging problems early, especially among older adults and people living with chronic or complex conditions.

While the review highlights key mechanisms and nursing strategies for maintaining continuity of care, contemporary challenges may influence their effectiveness. For example, the increasing digitalization of health records and communication systems can both facilitate and complicate relational continuity, depending on usability and integration. Similarly, nursing workforce constraints and workload pressures may limit the consistent implementation of transitional care interventions, potentially affecting patient outcomes. Acknowledging these tensions underscores the need for context-sensitive strategies that adapt continuity mechanisms to current healthcare environments.

Practical implications for nursing and service delivery include: (1) assigning clear responsibility for post-discharge follow-up, (2) ensuring discharge information is complete, timely, and usable by community providers, (3) providing patients and caregivers with a clear, plain-language plan for medications, red flags, and appointments, and (4) using structured follow-up contacts (for example, phone calls or home visits) to confirm understanding and address barriers to implementation.

Clinical illustration. Consider a 32-year-old patient discharged after an acute psychiatric admission with changes in medication and a recommended community follow-up within 7 days. If the discharge summary is delayed or incomplete (informational continuity), if responsibility for follow-up is unclear between hospital and community teams (management continuity), and if the patient does not have a consistent contact person (relational continuity), the transition may be experienced as fragmented and unsafe—leading to missed appointments, poor adherence, and early relapse. In contrast, a clear written plan, same-week outreach by a designated nurse or case manager, and a reliable channel for questions can transform the same transition into a coherent, supportive experience.

Finally, continuity challenges may be amplified in health systems facing geographic and resource constraints, such as rural or peripheral regions [5]. Strengthening community-based nursing services and interprofessional collaboration is likely to be critical for both quality and equity.

Together, these findings suggest that continuity of care should be understood not only as an organizational goal but as a dynamic relational process co-created by patients, professionals, and healthcare systems. Strengthening informational, management, and relational continuity may represent a key pathway toward safer and more patient-centered care transitions. It should be noted that the mechanisms and effectiveness of continuity interventions may differ across health system contexts, including variations in resource availability, care models, and regional healthcare infrastructure. These contextual factors may influence the generalizability of the findings and highlight the importance of tailoring continuity strategies to specific system environments.

### 4.1. Limitations

As a narrative review, this paper did not follow a formal systematic search protocol and therefore cannot claim to have identified all relevant publications on continuity of care and care transitions. The literature is heterogeneous in terms of populations, settings, definitions, and outcome measures, limiting direct comparability between studies and precluding quantitative synthesis. In addition, narrative reviews are subject to potential selection bias: study inclusion and interpretation depend on author judgment, and prioritizing highly cited or seminal frameworks may lead to over-representation of prominent literature while under-representing newer or less visible evidence. Finally, much of the included evidence emphasizes older adult populations and specific health-system contexts, which may limit generalizability to other groups and settings.

### 4.2. Future Directions

Future work should more explicitly align continuity dimensions with measurement and intervention design. First, there is a need to validate and implement patient-reported continuity tools (e.g., PCCQ/CAHPS-derived domains) across languages and settings, and to link continuity PREMs/PROMs to clinical and utilization outcomes in longitudinal designs. Second, mechanistic research should test how relational continuity—through trust and sustained engagement—mediates the relationship between informational/management continuity and post-discharge outcomes over time. Third, intervention research would benefit from comparative and component-based approaches (e.g., factorial or pragmatic designs) to identify which elements (medication reconciliation, early outreach, named contact person, shared care plans) drive improvement for which risk strata and contexts. Fourth, future studies should address under-studied populations, including younger patients with complex needs and mental health transitions, where discontinuities in accountability and follow-up may be especially consequential. Finally, hybrid effectiveness–implementation evaluations are needed to assess feasibility, workforce burden, and integration with digital infrastructure (EHR interoperability, automated discharge summaries, proactive outreach triggers) to support scalable continuity improvements in real-world systems.

## 5. Conclusions

Continuity of care across hospital-to-community transitions should be approached as a multidimensional process spanning informational, management, and relational continuity. Given the heterogeneity of study designs, outcomes, and contexts in the reviewed literature—and the interpretive nature of a narrative synthesis—practice implications should be applied cautiously and tailored to local system constraints and patient risk profiles. Nurses often play key roles within multidisciplinary transition efforts (e.g., discharge education, coordination, and follow-up), alongside physicians, pharmacists, and community teams; the relative contribution of specific professional models is likely to vary by system design and implementation. Strengthening continuity will therefore require coordinated, context-sensitive strategies that clarify accountability, improve information transfer, and support sustained therapeutic relationships after discharge.

## Figures and Tables

**Table 1:** Overlapping constructs and how they contribute to continuity of care during hospital-to-community transitions.

Primary Continuity Dimension	How It Contributes to Continuity?	Brief Definition	Term
Informational; Management	Improves completeness and timeliness of transition information and clarifies immediate next steps after discharge	Structured preparation for leaving hospital (information transfer, medications, follow-up arrangements, education, readiness)	Discharge planning
Management; Informational	Reduces fragmentation by aligning roles, responsibilities, and follow-up across settings	Organizing care activities across providers/services to ensure a coherent plan and clear accountability	Care coordination
Management; Informational; (often) Relational	Supports implementation and adaptation of the care plan after discharge; reduces early post-discharge gaps	Time-limited set of practices bridging hospital-to-community transitions (e.g., structured discharge, medication reconciliation, proactive follow-up)	Transitional care (programs/models)
Management; Relational	Strengthens continuity by improving follow-through, reducing barriers, and maintaining a consistent “anchor” during transitions	A consistent point of contact (often nurse-led) who supports patients in moving through complex systems and accessing services	Navigation and care coordination roles
Management (system-level); Informational	Creates system-level structures that enable coherent care journeys for patients receiving services from multiple providers over time	Multi-level coordination across organizational/professional boundaries to reduce fragmentation (clinical–organizational–system integration)	Integrated care approaches
Relational; Management	Supports ongoing follow-up and coordination in the community, reinforcing longitudinal therapeutic relationships	Primary care models emphasizing team-based care, proactive management, enhanced access, and sustained relationships	Patient-centered models in primary/community care (e.g., Patient-Centered Medical Home—PCMH)
All three (cross-cutting assessment)	Complements administrative outcomes by measuring continuity as lived experience (including coordination/communication domains)	Tools capturing whether care was experienced as coherent, coordinated, respectful, and responsive	Patient experience and patient-reported measures (e.g., PCCQ; CAHPS/CG-CAHPS domains)

Continuity dimensions: **informational**, **management**, and **relational continuity**. Note: Mapping reflects interpretive synthesis; constructs may overlap across continuity dimensions.

## Data Availability

No new data were created or analyzed in this study. Data sharing is not applicable to this article.

## Abbreviations

The following abbreviations are used in this manuscript:

CAHPS	Consumer Assessment of Healthcare Providers and Systems
CG-CAHPS	Clinician & Group Consumer Assessment of Healthcare Providers and Systems
CINAHL	Cumulative Index to Nursing and Allied Health Literature
NHS	National Health Service
PCCQ	Patient Continuity of Care Questionnaire
PCMH	Patient-Centered Medical Home
